# Supplementary material for: Non-replicating adenovirus based Mayaro virus vaccine elicits protective immune responses and cross protects against other alphaviruses
Source: PLoS Negl Trop Dis. 2021 Apr 1;15(4):e0009308. doi: 10.1371/journal.pntd.0009308 (PMC8051823; doi:10.1371/journal.pntd.0009308)
Supplement: Attachment — Submitted filename: PNTD-D-20-01861-Review.docx [file pntd.0009308.s003.docx]

Manuscript Number: PNTD-D-20-01861

Full Title: Non-Replicating Adenovirus Based Mayaro Virus Vaccine Elicits Protective Immune Responses and Cross Protects Against Other Alphaviruses

The research paper provides a great insight on the generation prophylactic MAYV vaccine expressing the complete viral structural polyprotein in a non-replicating human adenovirus V (AdV) platform. AdV-MAYV vaccine were tested for generation of immune responses, cross-neutralizing antibodies against two related arthritogenic alphaviruses-CHIKV and Una viruses in mice and then challenged for preventing viremia, reducing viral dissemination to tissues and mitigating viral disease in IFN⍺R1^-/-^ mice. Furthermore, they have reported that immune serum from the from vaccinated animals were able to prevent infection and diseases, indicating that antiviral antibodies are protective. Based on the data, they concluded that these cross-neutralizing antibodies were protective against lethal infection in mice following challenge with these heterotypic alphaviruses. The paper is well written, and the experimental design is good including all the necessary control groups.

This approach has potential implications for development of an MAYV vaccine and may also have applications for other mosquito borne or other Alphavirus viruses’ pathogeneses. This approach also demonstrates interesting biologically phenomena and prophylactic MAYV vaccine responses in mouse model. The scope of this paper is too broad, and the data may be sufficient to make a conclusion. The paper would be acceptable after addressing some of the issues described below.

1. How far the AdV-MAYV booster immunization would be effective or long lasting in mediating immune response, both humoral and cell mediate.
2. Functional studies may require enumerating the epitope binding predictions of AdV-MAYV E1 and E2 as a candidate vaccine.
3. It would have been nice to see if each separate antigen and T cell dependent/independent would improve the immune response against it.
4. Figure 1, please add a sentence to clarify whether other E1, E2 and 6k is present in mature virions of as implied. These proteins appear to be involved in efficient virus budding. No expression data.
5. For the neutralization assay should contain diluted irrelevant serum for control. Most of the Alphaviruses can be neutralized to a degree by non-immune mouse serum and this should be considered in neutralization assays.
6. Also, the authors not adequately included the correct references.

**Reviewer's Responses to Questions**

**Methods**

-Are the objectives of the study clearly articulated with a clear testable hypothesis stated? Yes

-Is the study design appropriate to address the stated objectives? Yes

-Is the population clearly described and appropriate for the hypothesis being tested? Yes

-Is the sample size sufficient to ensure adequate power to address the hypothesis being tested? Yes

-Were correct statistical analysis used to support conclusions? Yes

-Are there concerns about ethical or regulatory requirements being met? Yes.

**Results**

-Does the analysis presented match the analysis plan? Yes

-Are the results clearly and completely presented? Yes

-Are the figures (Tables, Images) of sufficient quality for clarity? Yes

**Conclusions**

-Are the conclusions supported by the data presented? Yes.

-Are the limitations of analysis clearly described? Yes.

-Do the authors discuss how these data can be helpful to advance our understanding of the topic under study? Yes.

-Is public health relevance addressed? Yes.
