# Supplementary material for: Non-replicating adenovirus based Mayaro virus vaccine elicits protective immune responses and cross protects against other alphaviruses
Source: PLoS Negl Trop Dis. 2021 Apr 1;15(4):e0009308. doi: 10.1371/journal.pntd.0009308 (PMC8051823; doi:10.1371/journal.pntd.0009308)
Supplement: Attachment — Submitted filename: AdV-MAYV vaccine manuscript - Reviewer responsesv3.docx [file pntd.0009308.s004.docx]

Manuscript Number: PNTD-D-20-01861

Full Title: Non-Replicating Adenovirus Based Mayaro Virus Vaccine Elicits Protective Immune Responses and Cross Protects Against Other Alphaviruses

We would like to thank the reviewers for their critical assessment of our manuscript. We are excited that the reviewers found the work interesting and well done. We have addressed the concerns raised by the reviewers and have made changes to the manuscript based upon their suggestions. Please find our remarks to their critiques below:

1. How far the AdV-MAYV booster immunization would be effective or long lasting in mediating immune response, both humoral and cell mediate.

**We tested the longevity of the immune protection and found that it lasts for at least 84 days post vaccination, with no signs of reduction at that time. Future longevity studies could extend out further but would require substantial time to complete.**

2. Functional studies may require enumerating the epitope binding predictions of AdV-MAYV E1 and E2 as a candidate vaccine. It would have been nice to see if each separate antigen and T cell dependent/independent would improve the immune response against it.

**We agree that it would be interesting to be able to isolate the protective nature against each antigen and plan to do this in future studies; it is of special interest to identify the cross-protective epitopes.**

3. Figure 1, please add a sentence to clarify whether other E1, E2 and 6k is present in mature virions of as implied. These proteins appear to be involved in efficient virus budding.

**The entire MAYV structural protein was cloned into the Adenovirus vector, which for Alphaviruses can result in the production of virus like particles with the full complement of structural proteins. We have added a sentence to indicate that we expect all of the structural proteins to be present in the VLP.**

4. For the neutralization assay should contain diluted irrelevant serum for control. Most of the Alphaviruses can be neutralized to a degree by non-immune mouse serum and this should be considered in neutralization assays.

**While we agree with the reviewer that there can be some low-level neutralization at high levels of serum, for our experiments we also included serum samples from animals that were infected with a control adenovirus such as GFP in order to get at this point.**

5. Also, the authors not adequately included the correct references.

**We have looked through the references as requested.**

6. Why do you have to add both High and Low viscosity CMC on infected cell monolayer?

**In general, Low viscosity CMC is not sufficient to prevent viral spread on cell monolayers; and High viscosity is more difficult to work with. Thus, we find that an equal mixture of High and Low offers a happy medium with good activity and easier workability. Perhaps switching to Intermediate viscosity CMC maybe the appropriate “happy medium” but this will require proper vetting in the future.**

7. For amplification of MAYV gene, before the PCR product amplification, you need the cDNA production, since you are working with an RNA virus. Please include this comment.

**We apologize to the reviewer that it was confusing about whether we produced cDNA prior to detection. This has been modified for clarity in the materials and methods section.**

8. For viral feet challenge, why did you use 10-log reduced viral load for CHIKV (10^3 PFU) compared to MAYV and UNAV?

**While we’ve been using CHIKV at 1e3 PFU for a while, we used data mined from the literature to establish the dosage for MAYV and UNAV.**

9. It is mentioned that you used 500uL of PBS for tissue harvest. If you used the same PBS volume for all collected tissue samples, I assume they would present different tissue concentrations, depending on sample size/mass. How did you normalized your PFU/qRT-PCR and cytokines results? I see that your tissue viral loads (PFU/mL and copies/mL) and cytokines quantification (pg/mL) are all in mL of tissue homogenate.

**Yes, all tissues were collected into the same volume of PBS and then homogenized by bead beating. These samples were used for cytokine, PFU, and vRNA assessments. We typically do not compare between tissue types only within a specific type. Thus, the samples are controlled within specific tissue type.**

10. Which frequency and equipment did you use for tissue homogenization? Please include this information

**The information has been added to the mouse experiments section of the materials and methods.**

11. 0.2% methyl blue': In Viruses section it is described '0.5% methylene blue'. Please, double-check

**The concentration was 0.2% and has been modified in the Viruses section.**

12. The MAYV reverse primer does not anneal in a reverse way to the target region. The complementary reverse sequence would be '5- CTTCCAGGCTGCCCGGCACCAT -3'. Please, double-check the sequence;

**We apologize the sequence was reverse/complement and has been fixed.**

13. "Cells were harvested 72 hpi after washing with PBS and were pelleted at 9,391 x g for 15 minutes at 4C." At this centrifugal g force I would expect a degree of cell lysing. Please, double-check

**The reviewer is correct and this mistake has been fixed in the materials and methods section.**

14. In the case of this experiment, the ideal PRNT positive control would be sera from MOCK infected mice instead of virus only. This sera may have unspecific or natural antibodies that may interfere in the viral neutralization and should better work as a background control.

**Sorry if this wasn’t clear, for negative controls we used serum from animals that were either mock infected or vaccinated with control AdV. Positive control sera was obtained from mice that were infected with Mayaro virus but not vaccinated.**

15. ELISA coating is typical performed with a basic buffer (pH > 9) such as Carbonate-bicarbonate buffer. Please, double check which buffer did you use and insert a trustful reference of ELISA with total Alphavirus antigens: 10.1371/journal.pntd.0000753

**We dilute our antigen in PBS and have had very good binding. But we will try this in the future to assess whether we get better binding. Thank you for this comment.**

16. Did you washed your plates after viral adsorption? Include this information;

**Yes, following viral adsorption the plates were blocked with PBS/milk and then washed 3 times with ELISA buffer. This information has been added to the materials and methods section.**

17. How much volume did you use for blocking? Include this information

**This information has been added to the materials and methods section.**

18. Did you evaluated your samples in duplicate, triplicate wells...? Include this information;

**No, we only compared biological replicates. This information has been added.**

19. What is the composition of 'ELISA wash buffer' and 'Elisa buffer' ?

**This information has been added.**

20. Please, give more information about the secondary antibodies used in these experiments;

**This information has been added.**

21. Did you not sliced, macerated and digested the spleen before cell strainer? It is quite a hard tissue to simple pass through the mesh. Please, double-check

**The rubber end of a 3ml syringe plunger works well to macerate spleen tissue and pass it through the mesh filter, this information has been added.**

22. Is there any specific reason to use 2ul volume of DMSO as the control? I suppose it should be the same volume used for the peptides

**As pointed out by the reviewer, this is an equivalent volume as the peptide used in the assays.**

23. Please include the ELISpot plate reader model and manufacturer;

**This information has been added to that section.**

24. You cannot properly cut 5-um sections of tissues that contains bone and cartilages. Did you decalcified and dehydrated the tissue? Removed the skin? Please, include a better description of this methodology and include references.

**Additional details have been added to the materials and methods section.**

25. What do you mean by 'severity of histological lesions'? Infiltrates? Necrosis? Hemorrhage? Tissue or cartilage destruction? This point should be clarified for better understanding.

**We have modified this section to make it clearer to the reader as to what the severity of histological lesions means.**

26. Figure 3B. Why are different bands detected with mouse serum compared to CHIKV Abs

**These bands represent the different forms of the envelop proteins due to differences in cleavage and glycosylation**.

27. Figure 5: The authors should comment on the Eotaxin values. Apparently, AdV-MAYV vaccination inhibits eotaxin compared to the untreated control.

**We agree that the slightly lower levels of Eotaxin are interesting but because it is not statistically significant, we do not want to over interpret that finding. It is unclear whether this difference would be biologically relevant.**

28. What is the Anti-alphavirus capsid antibody? Please describe in Methods or in the figure legend, if it is a commercial poly/monoclonal antibody or an in house developed antibody.

**We have cited the mAb targeting alphavirus nucleocapsid proteins.**

29. In the VLP electronic microscopy (Fig 1C), do you have another evidence that those viral particles are the Alphavirus VLPs and not the Adenoviruses?

**While we cannot discount that there might be some residual AdV we would not expect that there would be enough to robustly detect by EM. The human fibroblasts cells used for the production of VLP were initially infected with the replication defective adenovirus, extensively washed with PBS.**

30. Fig 2C: How do you explain the neutralization activity of Control Serum group? Please comment;

**We are not sure why this happened with that sample. The PBS control mouse serum did not show any neutralization, which is the better control. Also the effect was not affected by dilution, which would be expected if the activity were specific.**

31. In Fig 3B, panel 4, we can see a different pattern of proteins recognition from vaccinated mice that differs from monoclonal antibodies but present some comparable molecular weight. I guess it would be careful only to assume that the antibodies from vaccinated mice probable are recognizing Envelope proteins considering the molecular weight size. Additionally, what evidence do you have that the lower band in Panel 4 is the capsid protein?

**That is a good question; we used purified virus preparations for this analysis and the protein observed is the correct size for viral capsid and similar to what was shown in figure 1 panel b. This band is not observed for antibodies directed against the viral envelope proteins.**

32. Please, give more information about the monoclonal antibodies (87H1 and 133.B4) in Methods, westblotting section

**Information regarding these antibodies have been added to the materials and methods section.**

33. Is the Supplementary figure 3 basically showing the same data of Figure 7A? Why do you consider necessary to additionally show it?

**The data shown in Supplementary Figure 3 was our initial analysis to determine whether IFNaR-/- mice would generate neutralizing antibodies after vaccination with AdV-MAYV. This experiment was separate from the data shown in Figure 7. We agree with the reviewer and have removed the supplemental figure 3 as the data is redundant.**

34. In the conclusion of this section, I would suggests to include that the results also demonstrate that antibodies are not the single mediators of viral protection, since you have a slight body weight reduction and detectable viremia development in serum (2dpi) and Ankle at (7dpi) in mice that received the sera

**We appreciate this comment and have added a conclusion that antibodies are not the sole mediator of protection.**

35. "MAYV infection causes a febrile illness for 3-5 days, while joint and muscle pain can last for up to a year. Based on the similarity of disease symptoms between Old-world alphaviruses and serological cross-reactivity, infections with MAYV are commonly misdiagnosed [3]." This phrase is almost the same as in the Introduction;

**We have cleaned up the discussion by deleting redundancy and we thank the reviewer for pointing this out.**

36. "Passive transfer experiments demonstrated robust and transferrable antibody production in AdV-MAYV vaccinated mice, protecting recipient mice from viremia at 2 dpi and enabling survival until study endpoint". The passive sera transfer experiment relate to virema detection was performed on IFNAR1-/- mice, which demonstrated reduced viremia when compared to non-vaccinated mice. Thus, I think this phrase should be "Partially protected from virema at 2 dpi".

**We agree with the reviewer and have made the suggested modification.**

37. "Our findings agree with these data and also suggest the spleen is a reservoir permissive to high levels of MAYV replication in the absence of innate immunity." Please, show me which of your data demonstrate the spleen as potential viral reservoir in the absence of innate immunity?

**We agree with the reviewer and have changed reservoir to Target of viral infection and added reference to Figure 4 to the end of that sentence.**

38. "The novel AdV-MAYV vaccine approach reported here is the fourth reported vaccine approach against MAYV and is the only MAYV vaccine approach that has shown the ability to cross-protect against both CHIKV and UNAV [8, 10, 31, 60]." Why do you claim to be the fourth reported vaccine candidate?

**We have updated this section to highlight our overall intent, which was to say that the AdV MAYV vaccine protects against CHIKV.**

39. Why is the cross-protection of UNA less efficient than that of CHIKV. UNA is closer related to MAYV. Please explain or comment.

**We believe that the *in vivo* protection against UNA in IFNaR-/- mice was equivalent to the protection against CHIKV, which includes mortality, weight loss, serum viremia and tissue viral loads.**

40. Figure 6B: No infection is indicated in the figure legend, but the columns are not visible.

**We have amended the figure axis to set at -1 and non-infection is clarified as the baseline control.**

41. Use of the phrase urban spread and mentioning urban transmission cycles is a little misleading. Isn’t this incriminated but not proven?

**We have softened the wording around this section as the reviewer is correct the urban cycle is a potential means of transmission especially in light of the ability of the virus to infect *Aedes* mosquitoes that can transmit the virus.**

42. Clarify if immunization was intramuscular in the methods here.

**Immunizations were delivered into the thigh muscle, this has been clarified in the text.**

43. Rationale for using the CHIKV Sri Lankan strain and not an American strain.

**We used the CHIKV Sri Lankan strain in order to compare our results to our previous studies in mice. While there are subtle nuances between these virus strains the overall serotype conservation makes the results applicable to other strains.**

44. There is no reference to sample size in these mouse experiments

**The mouse sample sizes are included in each of the figure legends.**

45. Rationale for using the strain of BeArr505411. Is this a genotype L strain?

**We used BeArr505411, which is a L genotype. Interestingly, we have compared the protein sequence for the structural proteins for the different genotypes and they are highly conserved making our neutralization data highly applicable to the other MAYV genotypes similar to what has been observed for CHIKV.**

46. There is no reference for safety studies of this vaccine. Can you discuss the safety of the vaccine in some regard?

**We did not observe any changes in mouse behavior, weight loss or other attributes that would indicate that the AdV has safety concerns for the dose and route that we are using it at. We have added this information to the text.**

47. Figure 6. passive is spelled incorrectly in the legend

**This has been fixed.**

48. Authors should describe how the PRNT50s remain comparable between the wt and IFN alpha KO mice

**This has been mentioned in the results section.**

49. The authors don’t discuss the implication of detecting virus in the ipsilateral ankle and its potential impact of arthralgia in the cross-protection studies.

**This has been discussed in results section.**

50. Discussion comparing the NT titers with this vaccine and other mayv vaccines would be useful

**We agree that experimentally determining the levels of protection provided by other MAYV vaccine candidates would be very helpful for comparing vaccine immunogenicity and efficacy. In addition, it might be helpful to then layer on that prime boost schedules that intermix the different types of vaccines. While this is of interest to us, this is currently beyond the scope of the current manuscript.**

51. Address the limitations of your cell-mediated response study.

**Our study was not aimed at defining all of the T cell epitopes present in mice that were vaccinated vs. infected. We feel strongly that this is an important goal, it would require a complete peptide library and more time to accurately identify all of the CD4 and CD8 epitopes targeted. This would be something that we would like to pursue in vaccinated non-human primates in the future.**

52. However, to date there are no approved vaccines against any alphaviruses. Previous MAYV vaccination attempts have included live-attenuated virus and DNA based vaccines [8-11]." Please include 'inactivated vaccine'

**This has been added as suggested.**

53. "... The infected cells were rocked continuously..." Suggestion: For viral adsorption, the cells were continuously rocked..., "...containing confluent monlolayers of Vero cells..."  Suggestion: 'Monolayer'; "... were fixed with 3.7% formalin diluted in 1x PBS." Suggestion: Remove '1x', and "Membranes were washed and then developed with ThermoFisher Pico". Suggestion: Substitute 'developed' by revealed; "One milliliter of 5% FBS/DMEM/CMC was added to each..." Suggestion: Substitute here and elsewhere '5% FBS/DMEM/CMC' by 'CMC DMEM media containing 5% FBS'; "...to each well and the plate was incubated for 48 h." Suggestion: include 'at 37℃ and 5% CO2 atmosphere'; "...3.7% formaldehyde to each well for 15 minutes, washed with cold water...". Suggestion: Include 'then the supernatant was removed and the wells were washed with cold water...'; "...and stained with 0.2% methyl blue dye for 15 minutes. Plates were washed..." Suggestion: Include: 'The dye was removed and the plates were washed...'; '...30 minutes and 9.4x104 PFU in 100 μl PBS was...' Suggestion: '...MAYV was diluted in PBS to 9.4x10^4 PFU/100uL...'; '...blocked for 1 h with 5% milk in 1X TBS with 1%...' Suggestion: Remove '1x'; 'Plates were then washed three times with ELISA buffer, blotted dry and developed with OPD substrate.' Suggestion: '...and colorimetric signal was developed...'

**Changes have been made as suggested.**

54. 'The Mayaro (MAYV)...' Suggestion: The Mayaro virus (MAYV); 'THF-CAR cells were infected with a multiplicity of infection (MOI) equal to 100 PFU/cell...' Suggestion: THF-CAR cells were infected with a MOI equal to 100 PFU/cell; "...that pre-formed CD8+ T cell responses have on alphavirus disease, and therefore, next determined whether AdV-MAYV..." Suggestion: ...we next determined...;"...receptor knockout (IFN⍺R1-/-) mice vaccinated with AdV-MAYV and the cells were plated onto IFN- ELISpot plates..." Suggestion: ...mice vaccinated with AdV-MAYV. The cells were...; "Epitopes in E2 have been identified as prominent targets of early T cell responses in CHIKV infection and support the N-terminus of E2 as an important target of the early adaptive immune response [38, 44]." This phrase should be moved to discussion;

**These have been changed as suggested.**

55. Title section suggestion: ...Vaccine Elicits Protection and Reduces Inflammatory...;

**We prefer to keep the title as it is unless the editorial staff feels that would be important.**

56. "infectious virus was undetectable in any of the tissues from both groups of AdV-MAYV vaccinated mice but virus was present in all tested tissues from..." Suggestion: ...AdV-MAYV vaccinated mice in contrast all tested...

**We have amended this sentence to clarify it.**

57. "In fact, for most chemokines there was no statistical difference in chemokine levels between AdV-MAYV vaccinated..." Suggestion: In fact, for all chemokines...

**This has been addressed in the modified version.**

58. "A previous study by Webb et. al., in which mice vaccinated with a CHIKV/IRES vector, as well as CHIKV infected mice, produced MAYV cross-reactive antibodies, implicated shared antigenic epitopes between MAYV and CHIKV [31]." Suggestion: ...produced limitated MAYV cross-reactive antibodies, indicating the existence of common antigenic epitopes.; Figure 4. "mice were challenged with 104 PFU/ml MAYVBeAr in the right footpad." Suggestion: Change PFU/ml to total PFU; Follow the figure pattern and add the red dots to Fig 4H

**We have made these modifications as suggested.**

59. Supplemental Figure 2. Please, double check the objective magnification. They all seems not to be right. Also, please include a properly scale bar information;

**We have added the magnification power to the figure legend.**

60. A comprehensive paper on a disease that is very minor with only 30-100 cases per annum (Nat Rev Rheumatol. 2012 May 8;8(7):420-9). This is not enough to justify a vaccine, either commercially or on public health grounds as most of these infections are self resolving. That the virus might remerge to be more of a problem is possible (e.g. Pathogens. 2020 Sep 8;9(9):738; Int J Infect Dis. 2020 Mar;92:253-258), but not reality at this stage. The paper should make these issues clear e.g. case numbers, market size etc and explain that MAYV is currently not a viable target for a vaccine.

**We appreciate the reviewer’s candor on this matter and take it to heart.** **However, we do not feel that we have overstated the public health grounds for the development of a Mayaro virus vaccine. But we have tempered the introduction to reflect the current need. As the reviewer suggests, a vaccine against MAYV is not currently warranted but the aim of our study is to lay the groundwork for the quicker and more informed decisions about how this vaccine platform performs against MAYV, in case the virus were to undergo an epidemic. A further developed vaccine against EBOV, CHIKV or ZIKV may have reduced the impact of these recent viral epidemics. We also agree that the cross protection elicited by any Alphavirus vaccine, at this point, is academic but there are many groups that are currently trying to understand cross reactivity to develop broadly protective Alphavirus vaccines.**

61. Statistics has some problems as parametric tests are used throughout when clearly some of the data is non-parametric. Fig. 5 RANTES – no way do these data have equal variance. Fig 5, Eotaxin – clearly highly skewed data i.e. not normal distribution. Non parametric tests should be used.

**The statistical analysis has been updated.**

62. The discussion on IgG3 and E2 is not so compelling. Interplay between IgM and IgG2b needs a reference and how this is supposed to relate to IgG3 is unclear.  Suggest consider deletion.

**We agree and have deleted that sentence.**

63. The First part of the Discussion, up to “In this report” is introduction and needs to be moved.

**This has been altered as suggested.**

64. Should make clear 6J vs 6N mice (Lab Anim Res. 2017 Jun;33(2):119-123).

**We appreciate this comment and have clarified the mouse strain.**

65. Ref 38 is in RAG mice which is somewhat out of context here given the very clear data that CD8 T cells have no protective role under normal circumstances (Front Immunol. 2020 Feb 21;11:287).

**The protective role for T cells under normal infection conditions remains debated as they can have many different types of functions depending upon type and activation status. The reference was meant to indicate that we have performed these types of assays as previously described, and the sentence now reflects this.**

66. Joint and muscle reservoirs needs a reference and is misleading – these may be sites of replication (PLoS Pathog. 2017 Dec 27;13(12):e1006788) but reservoir implies something quite different.

**We have changed reservoirs to “targets”.**

67. “levels than infected mice.” Seems to have some words missing higher than what?

**We have modified this sentence.**

68. Fig.2B PRNTs might have some SDs added.

**The analysis was performed in Prism at a 95% confidence interval, accordingly we do not feel that SD or SE can be accurately derived from these data.**

69. “No approved vaccines against any alphavirus”.  This is incorrect a Getah vaccine is commercial available in Japan from Nisseiken (https://www.jp nisseiken.co.jp/en/products/pdf/horse/JE_GETA_en.pdf).

**We have added the Getah LAV to the introduction of the manuscript.**

70. “effective treatments” - NSAIDs are unquestionably an effective treatment, and are widely used.  Like all treatments for anything, they are not 100% effective, but they are nevertheless effective at ameliorating arthropathy.

**That is correct and we only argue that there are no specific treatments. We have tempered this statement to reflect this discussion.**

71. The claims regarding myalgia and arthralgia cannot be made as these terms are defined as pain in joints and pain in muscle, and there is no way pain can be measured in these mouse models.

**That is correct. Myalgia is a symptom and we have modified the text to indicate this.**

72. This work represents an innovative vaccine development approach for emergent viral diseases caused by MAYV, CHIKV and UNAV, which are a world public health concern specially in undeveloped countries. However, at the time of manuscript revision, it has been published elsewhere a manuscript that utilized the same vaccine strategy, describing an Adenoviral-Vectored MAYV and CHIKV vaccine candidate, which are able to confer partial cross-protection against viral infection in mice (10.3389/fimmu.2020.591885). Very similarly, their findings demonstrate that AdV-MAYV vaccine provides full protection (reduction of viremia, weight loss and foot swelling) against MAYV challenge with increased neutralizing antibodies production, which partially cross-protect against CHIKV. Please, I would suggest that these findinds should be commented and discussed in the manuscript.

**We have addressed this in the manuscript.**
